# Epidemiological Profile and Temporal Trends of Exogenous Poisoning in the State of São Paulo and Brazil From 2010 to 2020

**DOI:** 10.7759/cureus.119276

**Published:** 2026-11-07

**Authors:** Pedro Henrique Serra Carvalho Dos Santos, Leonardo De Felice Wolff, Vinícius Domingues Camurça, Felipe Lemos Martins, Rodrigo Ganem Sugino

**Affiliations:** 1 Faculty of Medicine, Centro Universitário Lusíada, Santos, BRA

**Keywords:** brazil, epidemiology, exogenous poisoning, public health surveillance, suicide attempt, time-series analysis

## Abstract

Background: Exogenous poisoning is a compulsorily notifiable condition in Brazil, but analyses of its temporal trends in the state of São Paulo remain scarce. This study aimed to describe the epidemiological profile of exogenous poisoning in the state of São Paulo from 2010 to 2020 and its distribution across Regional Health Care Networks (Redes Regionais de Atenção à Saúde (RRAS)) and to analyze temporal trends in incidence, mortality, and case fatality compared with Brazil.

Methods: An observational time-series study was conducted using data from the Notifiable Diseases Information System (Sistema de Informação de Agravos de Notificação (SINAN)), obtained through the TabNet platform of the Brazilian Ministry of Health, and population estimates from the Brazilian Institute of Geography and Statistics. Sociodemographic, exposure, and outcome variables were analyzed. Incidence, mortality, and case fatality rates were calculated, and notifications were stratified by RRAS. Temporal trends were assessed by joinpoint regression, estimating the annual percent change (APC) with 95% confidence intervals (CIs) for each segment and for the entire period, with sensitivity analyses.

Results: A total of 315,266 notifications and 3,209 deaths were recorded in São Paulo, accounting for 25.7% of notifications and 27.4% of deaths in Brazil. Cases predominated among females (n=169,740; 53.8%), individuals aged 20-39 years (n=142,989; 45.4%), and White individuals (n=166,896; 52.9%). Medications were the main toxic agent (n=144,567; 45.9%), and suicide attempt was the main circumstance (n=125,491; 39.8%). Incidence was 64.7 cases per 100,000 inhabitants per year (Brazil: 54.8), mortality was 6.6 deaths per 1,000,000 inhabitants per year (Brazil: 5.2), and case fatality was 1.02% (Brazil: 0.95%). Incidence (APC=13.9%; p<0.001) and mortality (APC=11.7%; p<0.001) increased over the period, whereas case fatality showed no significant trend over the entire period; a decline between 2016 and 2020 (APC=-14.8%; p=0.031) was not statistically significant in sensitivity analyses. The highest incidence was observed in RRAS 12 (São José do Rio Preto/Araçatuba; 126.2 per 100,000 inhabitants per year), a region of intense agricultural activity.

Conclusions: Exogenous poisoning in São Paulo showed increasing incidence and mortality, at levels above the national average, with a decline in case fatality after 2016 that should be interpreted with caution, and marked regional heterogeneity. These findings support strengthening toxicological surveillance and tailoring prevention strategies to regional and epidemiological characteristics.

## Introduction

Exogenous poisoning can be defined as a pathological process caused by substances that disrupt the body's balance through biochemical reactions and that may result in clinical manifestations, laboratory abnormalities, sequelae, and death [1]. It is a worldwide public health problem: World Health Organization (WHO) estimates indicate that, in 2019, approximately two million lives and 53 million disability-adjusted life years were lost due to exposure to selected chemicals worldwide, figures higher than those estimated for 2016 and 2012 [2]. Given its magnitude, exogenous poisoning has been included in the Brazilian National List of Compulsory Notification since 2010 [3], with data consolidated in the Notifiable Diseases Information System (Sistema de Informação de Agravos de Notificação (SINAN)).

Toxicological Information and Assistance Centers (Centros de Informação e Assistência Toxicológica (CIATox)) are specialized services that provide toxicological guidance to the public and to health professionals, contributing both to the clinical management of poisoning and to epidemiological surveillance [4]. The state of São Paulo hosts nine of the 32 CIATox in Brazil, including those in the cities of Campinas, Ribeirão Preto, Santos, and São José do Rio Preto, in addition to the state capital [5]. Despite this assistance network, the profile of exogenous poisoning notified in the state remains poorly characterized.

Between 2007 and 2019, Brazil showed increasing trends in the incidence and mortality of exogenous poisoning [6]. However, national estimates may mask important differences between states, and, to our knowledge, no study has analyzed temporal trends of notified exogenous poisoning in São Paulo, the most populous state in the country, or the distribution of cases across its regional health networks, which limits the planning of surveillance and prevention actions tailored to the state. A recent study showed that the configuration of the Regional Health Care Networks (Redes Regionais de Atenção à Saúde (RRAS)), the planning and regionalization units of the Brazilian Unified Health System (Sistema Único de Saúde (SUS)), influences the population's geographic access to health services, with heterogeneous effects across regions of the state [7]. This supports the use of RRAS as a unit for regional health analyses.

The objectives of this study were (1) to characterize the epidemiological profile of exogenous poisoning notifications in the state of São Paulo between 2010 and 2020, (2) to describe their distribution across the RRAS, and (3) to analyze temporal trends in incidence, mortality, and case fatality, comparing them with those observed in Brazil as a whole.

## Materials and methods

Study design and setting

This was an observational, ecological time-series study of exogenous poisoning in the state of São Paulo and in Brazil from 2010 to 2020, reported in accordance with the Strengthening the Reporting of Observational Studies in Epidemiology (STROBE) guidelines [8]. Exogenous poisoning became a compulsorily notifiable condition with the publication of Ministry of Health Ordinance GM/MS No. 2,472 of August 31, 2010, which made the reporting of all suspected poisoning cases mandatory nationwide [3]. The year 2010 was therefore defined as the starting point, and the study period covered the first 11 years of compulsory notification (2010-2020), thus covering the period from the implementation of compulsory notification up to the onset of the COVID-19 pandemic.

Study population and variables

The study population comprised all notified cases of exogenous poisoning in the state of São Paulo and in Brazil between 2010 and 2020. Sociodemographic variables (sex, age group, race/skin color, and education), exposure variables (toxic agent, circumstance, and type of exposure), and outcome variables (final classification, confirmation criterion, and outcome) were analyzed. Age was categorized into six groups: <10, 10-14, 15-19, 20-39, 40-59, and ≥60 years. Deaths were defined as notifications with the outcome "death from exogenous poisoning" recorded in SINAN. For the spatial analysis, notifications from São Paulo were stratified according to the 18 RRAS defined by the Bipartite Interagency Commission (Comissão Intergestores Bipartite (CIB)) in Deliberation No. 30/2024 [9].

Data sources

Data were extracted in September 2024 from TabNet, a free application provided by the Department of Informatics of the Brazilian Unified Health System (DATASUS), which gives access to the SINAN database [10]. The exogenous poisoning module (notifications registered in SINAN) was used, with tabulations by year of notification for 2010-2020, for Brazil and for the state of São Paulo (selected by state of notification), for the variables sex, age group, race/skin color, education, toxic agent, circumstance, type of exposure, final classification, confirmation criterion, and outcome. For the RRAS analysis, notifications from São Paulo were tabulated directly by RRAS in TabNet, according to the 18-RRAS configuration [9]. Because TabNet provides aggregated tabulations of consolidated SINAN data, in which duplicate notifications are handled by surveillance routines before consolidation, no additional record-level deduplication was possible. Population estimates used to calculate rates were obtained from the Brazilian Institute of Geography and Statistics (Instituto Brasileiro de Geografia e Estatística (IBGE)) [11].

The cartographic base of the state of São Paulo was also obtained from IBGE, and municipalities were grouped according to their respective RRAS. The spatial distribution was represented by a choropleth map produced with the GeoPandas library, version 1.1.4 (GeoPandas developers, open-source software; https://geopandas.org) [12], for Python, version 3.12.3 (Python Software Foundation, Wilmington, Delaware, United States). RRAS were shaded along a color gradient proportional to the incidence rate using linear normalization, so that the RRAS with the lowest rate corresponded to the lightest end of the color scale and the RRAS with the highest rate to the darkest end. The incidence rate of each RRAS was calculated as the number of notifications divided by the sum of the annual population estimates for 2010-2020 (person-years), multiplied by 100,000.

Statistical analysis

A descriptive analysis was first performed, with absolute and relative frequencies, using Microsoft Excel (Microsoft Corporation, Redmond, Washington, United States). Incidence rates (per 100,000 inhabitants per year) were calculated by dividing the number of notifications by the sum of the annual population estimates for the period (person-years), which corresponds to the average annual rate over the period. Mortality rates (per 1,000,000 inhabitants per year) were calculated by dividing the number of deaths by the same population denominator. Case fatality was calculated as the ratio between the number of deaths and the number of notifications, multiplied by 100. Annual rates were calculated using the population estimate for each year. Missing or unknown information ("ignored/blank") was kept as a separate category and included in the denominators of percentages and of case fatality. A sensitivity analysis of case fatality was performed under different assumptions about notifications with missing outcome.

Proportions between independent groups were compared using the two-proportion Z test, including the proportion of each toxic agent and circumstance between sexes and case fatality between males and females. Incidence rates across RRAS were compared using the same Z test, with each RRAS compared with the RRAS with the highest rate. To account for multiple testing, a Bonferroni-corrected significance threshold was applied to these 17 comparisons (p<0.003). For all other comparisons, statistical significance was set at p<0.05.

Temporal trends in the number of notifications, incidence, mortality, and case fatality were analyzed by joinpoint regression using the Joinpoint Regression Program, version 5.0.2 (Statistical Methodology and Applications Branch, Surveillance Research Program, National Cancer Institute, Bethesda, Maryland, United States) [13]. This method fits log-linear segments to the time series and identifies the points at which a significant change in trend occurs [14]. In accordance with the software's default for series with 11 data points, a maximum of one joinpoint was allowed. The annual percent change (APC) and its 95% confidence interval (CI) were estimated for each segment, and the APC for the entire period (2010-2020) was estimated from a log-linear model without joinpoints. Positive APC values indicated an increasing trend and negative values a decreasing trend; when the 95% CI included zero, the trend was classified as stationary. Three sensitivity analyses were performed: models with two joinpoints; the case fatality series for 2010-2019, excluding 2020, the first year of the COVID-19 pandemic; and case fatality in São Paulo recalculated under three assumptions about notifications with missing outcome (the same case fatality as notifications with a recorded outcome and 1.5 and 2 times higher). The model with one joinpoint was kept as the main analysis, given the small number of annual observations and the risk of over-parameterization. Analyses were performed using IBM SPSS Statistics for Windows, Version 26.0 (Released 2019; IBM Corp., Armonk, New York, United States), Minitab, version 21.2 (Minitab, LLC, State College, Pennsylvania, United States), and Microsoft Excel.

Ethical considerations

This study used only publicly available, aggregated, and anonymized secondary data from SINAN, accessed through TabNet/DATASUS, with no possibility of individual identification. In accordance with Brazilian National Health Council Resolution No. 510 of April 7, 2016, research using publicly accessible information and aggregated databases without individual identification does not require review by a research ethics committee.

Data availability

The data used in this study are in the public domain and available from SINAN through TabNet/DATASUS (http://tabnet.datasus.gov.br/cgi/tabcgi.exe?sinannet/cnv/Intoxbr.def). Population estimates are available from IBGE through TabNet/DATASUS (http://tabnet.datasus.gov.br/cgi/deftohtm.exe?ibge/cnv/popsvsbr.def).

Use of artificial intelligence 

Generative artificial intelligence (Claude; Anthropic PBC, San Francisco, California, United States) was used to assist in the translation of the manuscript into English, to improve its language and readability. All content was reviewed, verified, and approved by the authors, who take full responsibility for the manuscript.

## Results

Between 2010 and 2020, 315,266 notifications and 3,209 deaths from exogenous poisoning were recorded in the state of São Paulo, with a 4.5-fold increase in notifications and a 3.4-fold increase in deaths over the period. In Brazil, 1,226,134 notifications and 11,705 deaths were recorded in the same period. The state of São Paulo accounted for 25.7% of national notifications and 27.4% of national deaths.

Regarding the overall profile of notifications (Table 1), exogenous poisoning was more frequent among females (n=169,740; 53.8%), whereas most deaths occurred among males (n=2,034; 63.4%). The 20-39-year age group had the highest frequency (n=142,989; 45.4%), as did White individuals (n=166,896; 52.9%). Race/skin color was missing in 53,162 (16.9%) notifications and education in 124,400 (39.5%).

**Table 1:** Epidemiological characteristics of exogenous poisoning notifications in the state of São Paulo, Brazil, 2010-2020 (N=315,266)

Variable	Category	n	%
Sex	Female	169,740	53.8
	Male	145,478	46.1
	Missing/unknown	48	0.02
Age group (years)	<10	38,899	12.3
	10-14	14,730	4.7
	15-19	46,018	14.6
	20-39	142,989	45.4
	40-59	60,916	19.3
	≥60	11,687	3.7
	Missing/unknown	27	0.01
Race/skin color	White	166,896	52.9
	Mixed race (parda)	74,900	23.8
	Black	18,336	5.8
	Asian	1,511	0.5
	Indigenous	461	0.1
	Missing/unknown	53,162	16.9
Education	Illiterate	1,221	0.4
	Incomplete 1st-4th grade of elementary school	9,916	3.1
	Complete 4th grade of elementary school	6,120	1.9
	Incomplete 5th-8th grade of elementary school	30,029	9.5
	Complete elementary school	17,105	5.4
	Incomplete high school	28,893	9.2
	Complete high school	47,051	14.9
	Incomplete higher education	6,693	2.1
	Complete higher education	7,677	2.4
	Not applicable	36,161	11.5
	Missing/unknown	124,400	39.5
Toxic agent	Medications	144,567	45.9
	Drugs of abuse	68,867	21.8
	Food and beverages	21,097	6.7
	Household products	17,504	5.6
	Rodenticides	11,937	3.8
	Industrial chemical products	7,326	2.3
	Agricultural pesticides	6,324	2.0
	Household pesticides	2,862	0.9
	Cosmetics/personal hygiene products	2,184	0.7
	Veterinary products	1,788	0.6
	Toxic plants	867	0.3
	Metals	842	0.3
	Public health pesticides	435	0.1
	Other	7,363	2.3
	Missing/unknown	21,303	6.8
Circumstance	Suicide attempt	125,491	39.8
	Abuse	72,773	23.1
	Accidental	45,695	14.5
	Habitual use	19,622	6.2
	Self-medication	9,674	3.1
	Food or beverage ingestion	7,153	2.3
	Administration error	3,215	1.0
	Therapeutic use	3,202	1.0
	Environmental	2,018	0.6
	Violence/homicide	1,592	0.5
	Attempted abortion	521	0.2
	Inadequate medical prescription	184	0.1
	Other	2,946	0.9
	Missing/unknown	21,180	6.7
Outcome	Recovery without sequelae	233,449	74.0
	Recovery with sequelae	5,193	1.6
	Death from exogenous poisoning	3,209	1.0
	Death from other causes	734	0.2
	Loss to follow-up	7,909	2.5
	Missing/unknown	64,772	20.5
Total	-	315,266	100.0

Medications were the main toxic agent (n=144,567; 45.9%), followed by drugs of abuse (n=68,867; 21.8%). Suicide attempt was the most frequent circumstance (n=125,491; 39.8%). Most cases resulted from acute single exposure (n=182,522; 57.9%), and 233,449 (74%) recovered without sequelae. Overall, 216,504 (68.7%) notifications were classified as confirmed poisoning and 53,017 (16.8%) as exposure only, and the clinical criterion was the most frequent confirmation criterion (n=182,326; 57.8%). Although medications were the most frequently recorded agent, case fatality was highest in poisoning by agricultural pesticides (211/6,324; 3.3%).

The proportions of the main toxic agents and circumstances differed significantly between sexes (p<0.001 for all comparisons). Medications predominated among females (n=104,390; 61.5%), followed by drugs of abuse (n=17,375; 10.2%). This order was reversed among males, with drugs of abuse (n=51,485; 35.4%) more frequent than medications (n=40,151; 27.6%). Suicide attempt was more frequent among females (n=90,451; 53.3%) and abuse among males (n=52,276; 35.9%). Overall case fatality was higher among males (2,034/145,478; 1.4%) than among females (1,174/169,740; 0.7%; p<0.001).

Medications were the main toxic agent in all age groups, accounting for 16,353 (42%) notifications among children under 10 years and reaching their highest proportion among adolescents aged 10-14 years (n=8,564; 58.1%). Among children under 10 years, household products (n=9,622; 24.7%) were the second most frequent agent. From 10 years of age onwards, this position was occupied by drugs of abuse in all age groups. Accidental exposure predominated among children under 10 years (n=30,043; 77.2%), whereas suicide attempt was the most frequent circumstance in all other age groups, particularly among adolescents aged 15-19 years (n=24,528; 53.3%).

The profiles of the three main circumstances were also characterized. In suicide attempts, females (n=90,451; 72.1%) and individuals aged 20-39 years (n=63,049; 50.2%) predominated, and medications were the main agent (n=96,108; 76.6%); case fatality was higher among males (713/35,027; 2%) than among females (708/90,451; 0.8%; p<0.001). In abuse, the pattern was reversed: males predominated (n=52,276; 71.8%), the 20-39-year age group was again the most frequent (n=40,899; 56.2%), and drugs of abuse were the main agent (n=55,238; 75.9%); case fatality was also higher among males (939/52,276; 1.8%) than among females (287/20,486; 1.4%; p<0.001). In accidental cases, males predominated (n=24,171; 52.9%), cases were concentrated among children under 10 years (n=30,043; 65.7%), and medications were the main agent (n=14,650; 32.1%); case fatality was higher among males (75/24,171; 0.31%) than among females (33/21,517; 0.15%; p<0.001).

Over the entire period, the incidence rate was 54.8 cases per 100,000 inhabitants per year in Brazil and 64.7 in São Paulo. The mortality rate was 5.2 deaths per 1,000,000 inhabitants per year in Brazil and 6.6 in São Paulo. Case fatality was 0.95% in Brazil and 1.02% in São Paulo. Among notifications with a recorded outcome in São Paulo, case fatality was 1.28% (3,209/250,494). Assuming that notifications with missing outcomes had a case fatality 1.5 or 2 times higher than that observed among notifications with a recorded outcome, the overall estimate would increase to 1.41% and 1.54%, respectively.

The annual incidence rate (Figure 1) followed a similar pattern in the state and in the country throughout the period, peaking in 2019 and declining in 2020. In São Paulo, it rose from 22.4 per 100,000 inhabitants in 2010 to 110.9 in 2019 and was 91.3 in 2020 (Brazil: 24.4, 89.9, and 69.2).

**Figure 1:**
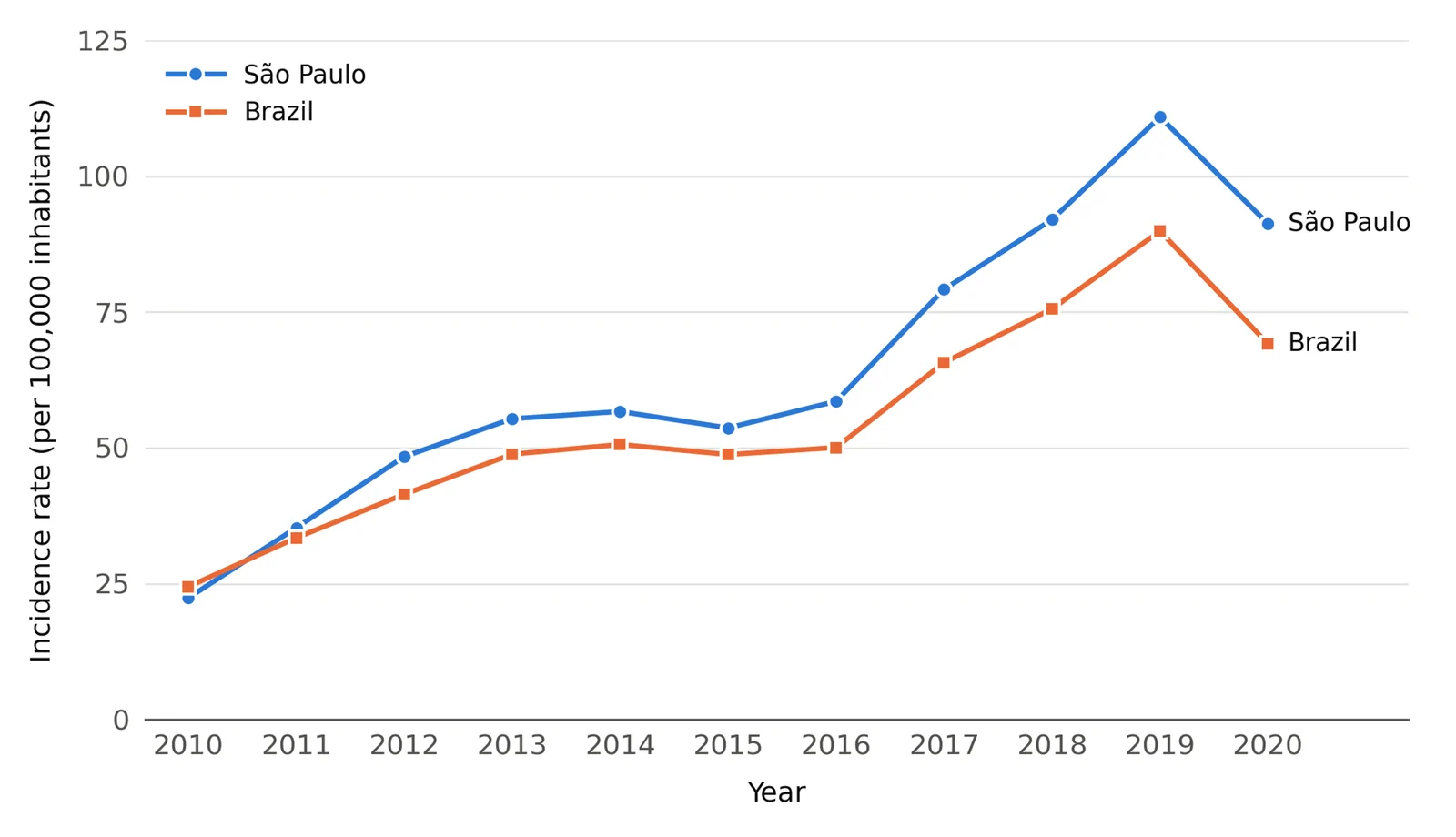
Annual incidence rate (per 100,000 inhabitants) of exogenous poisoning in the state of São Paulo (n=315,266) and in Brazil (n=1,226,134), 2010-2020

From 2014 onwards, the annual mortality rate (Figure 2) was higher in São Paulo than in Brazil. In São Paulo, it rose from 2.5 deaths per 1,000,000 inhabitants in 2010 to a peak of 9.5 in 2016 and was 7.8 in 2020 (Brazil: 3.3 in 2010, peak of 6.3 in 2018, and 4.9 in 2020).

**Figure 2:**
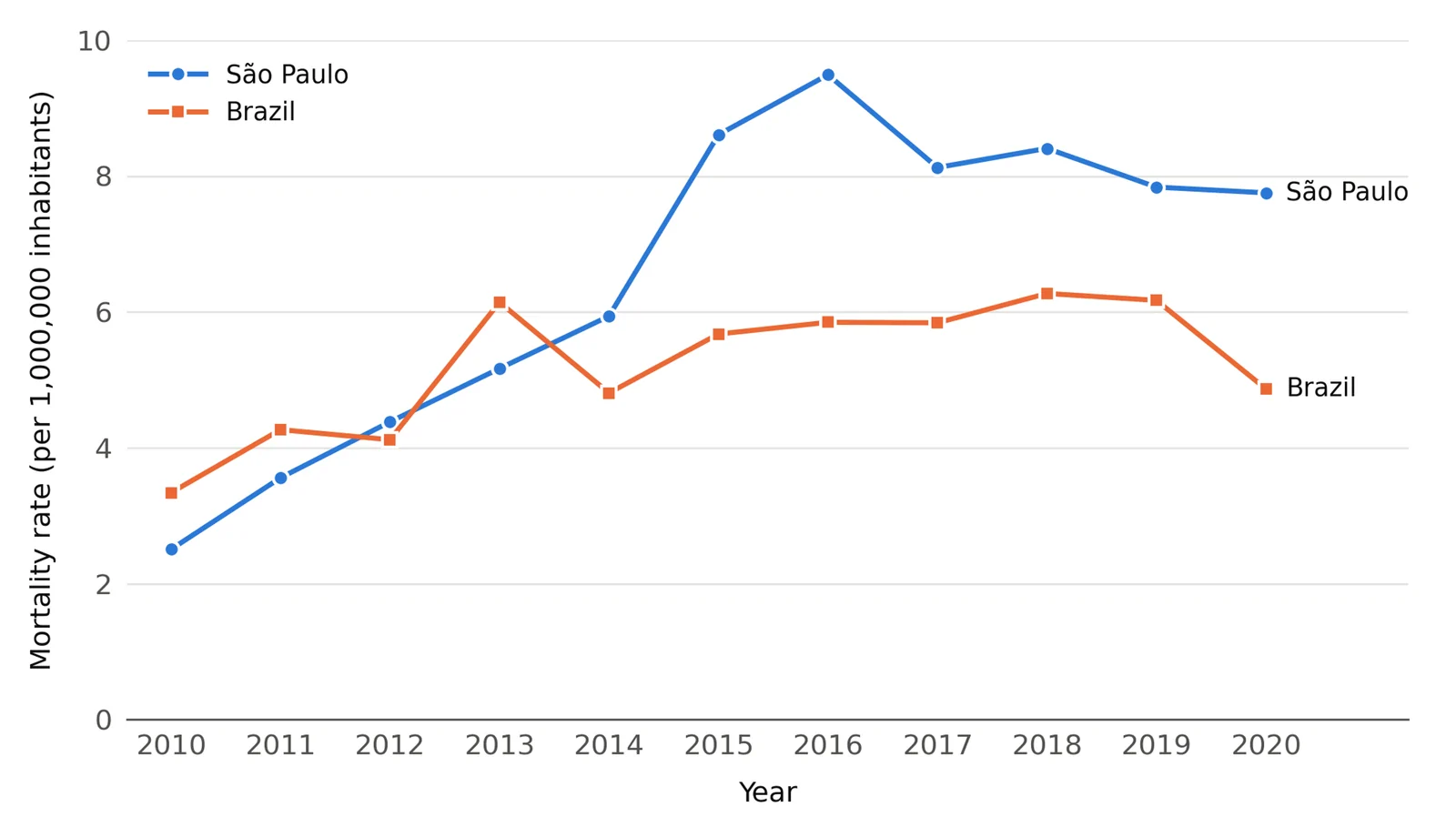
Annual mortality rate (per 1,000,000 inhabitants) of exogenous poisoning in the state of São Paulo (n=3,209) and in Brazil (n=11,705), 2010-2020

The spatial distribution of the incidence rate revealed heterogeneity among the states' RRAS (Figure 3). RRAS 12 (São José do Rio Preto/Araçatuba) had the highest rate (126.2 per 100,000 inhabitants per year), significantly higher than that of all other RRAS (p<0.001 for all comparisons), followed by RRAS 10 (Marília; 88.3 per 100,000), whereas RRAS 8 (Sorocaba) had the lowest rate (30.1 per 100,000). The city of São Paulo (RRAS 6) had the highest absolute number of cases (88,529) but ranked eighth when population size was taken into account (67.8 per 100,000).

**Figure 3:**
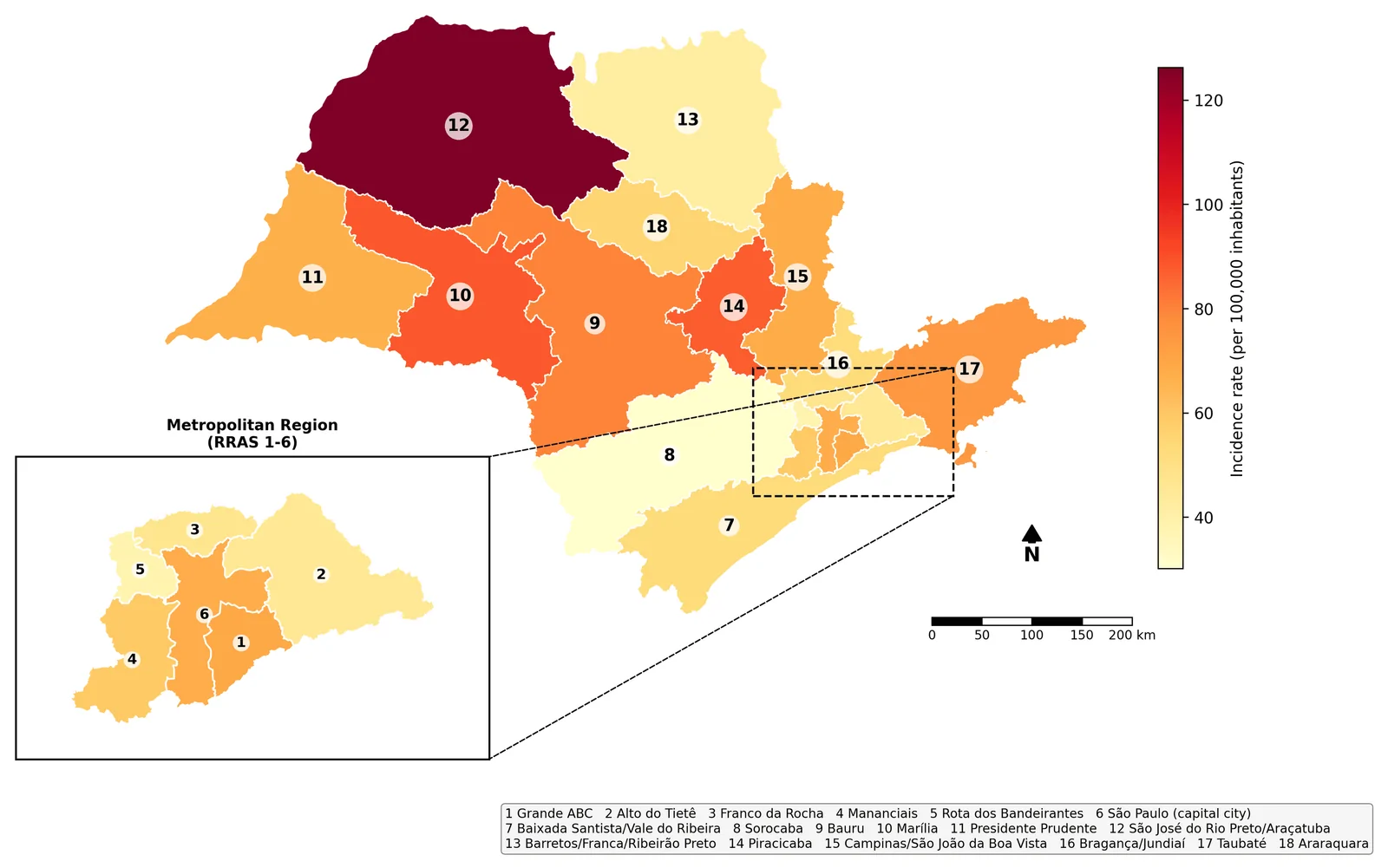
Incidence rate of exogenous poisoning (per 100,000 inhabitants per year) by Regional Health Care Network (Redes Regionais de Atenção à Saúde (RRAS)) in the state of São Paulo, 2010-2020 (n=315,266)

In São Paulo, joinpoint regression (Table 2) showed an increasing trend in incidence over the entire period (APC=13.9%; p<0.001), with a steeper increase between 2010 and 2012 (APC=42.3%; p<0.001) and a more moderate but significant increase between 2012 and 2020 (APC=10.2%; p=0.030). Mortality also increased over the entire period (APC=11.7%; p<0.001), concentrated between 2010 and 2016 (APC=23.1%; p<0.001), with a stationary trend between 2016 and 2020 (APC=-5.9%; p=0.106). Case fatality showed no significant trend over the entire period (APC=-1.9%; p=0.453) or in the first segment (2010-2016; APC=6.3%; p=0.093) but decreased significantly between 2016 and 2020 (APC=-14.8%; p=0.031). In Brazil, incidence increased over the entire period (APC=11.1%; p<0.001), with a stationary trend from 2012 onwards (APC=8.5%; p=0.068), whereas case fatality decreased (APC=-6%; p=0.004), particularly between 2016 and 2020 (APC=-11%; p=0.002).

**Table 2:** Temporal trends in notifications, incidence, mortality, and case fatality of exogenous poisoning in the state of São Paulo and in Brazil, 2010-2020 (joinpoint regression) Incidence per 100,000 inhabitants; mortality per 1,000,000 inhabitants; case fatality in %. APC: annual percent change; CI: confidence interval

Indicator	Location	Period	APC (%)	95% CI lower	95% CI upper	P-value	Trend
Notifications	Brazil	2010-2020	12.0	8.2	16.0	<0.001	Increasing
		2010-2012	31.3	13.1	48.8	<0.001	Increasing
		2012-2020	9.4	-0.4	12.3	0.056	Stationary
	São Paulo	2010-2020	14.9	9.5	20.8	<0.001	Increasing
		2010-2012	43.8	19.5	67.8	<0.001	Increasing
		2012-2020	11.2	3.5	14.0	0.029	Increasing
Incidence	Brazil	2010-2020	11.1	7.3	15.1	<0.001	Increasing
		2010-2012	30.1	12.1	47.6	<0.001	Increasing
		2012-2020	8.5	-1.3	11.5	0.068	Stationary
	São Paulo	2010-2020	13.9	8.5	19.7	<0.001	Increasing
		2010-2012	42.3	18.5	65.4	<0.001	Increasing
		2012-2020	10.2	2.8	13.0	0.030	Increasing
Mortality	Brazil	2010-2020	4.4	1.1	7.9	0.009	Increasing
		2010-2013	18.3	7.3	38.2	<0.001	Increasing
		2013-2020	0.4	-7.5	3.3	0.996	Stationary
	São Paulo	2010-2020	11.7	6.5	17.1	<0.001	Increasing
		2010-2016	23.1	17.9	31.5	<0.001	Increasing
		2016-2020	-5.9	-16.5	1.4	0.106	Stationary
Case fatality	Brazil	2010-2020	-6.0	-9.9	-1.9	0.004	Decreasing
		2010-2016	-3.0	-7.5	10.5	0.469	Stationary
		2016-2020	-11.0	-22.6	-5.3	0.002	Decreasing
	São Paulo	2010-2020	-1.9	-6.9	3.1	0.453	Stationary
		2010-2016	6.3	-1.6	50.8	0.093	Stationary
		2016-2020	-14.8	-40.2	-3.1	0.031	Decreasing

In the sensitivity analyses, the trends for the entire period were unchanged, and models with two joinpoints produced wide confidence intervals for most segments. The decline in case fatality after 2016 in São Paulo was consistent in direction in all sensitivity analyses but did not reach statistical significance in any of them: in the model with two joinpoints (2015-2020; p=0.095); when 2020 was excluded, in which the decline was steeper (2016-2019; APC=-20.9%; 95% CI -43.2 to 10.1; p=0.128); and when notifications with missing outcome were taken into account, in which its magnitude was attenuated (APC between -11.8% and -9.7%; p between 0.182 and 0.271). The proportion of notifications with missing outcome in São Paulo increased from 16.4% in 2016 to 24.8% in 2019.

## Discussion

Between 2010 and 2020, the number of notifications and mortality from exogenous poisoning increased in São Paulo at a faster pace than in Brazil as a whole. Notifications were more frequent among females, whereas deaths and case fatality were higher among males. In suicide attempts, case fatality among males was more than twice that among females. Medications were the most frequent agent, suicide attempt was the predominant circumstance, and agricultural pesticides were the most lethal agent.

Case fatality was higher among males in all three main circumstances of poisoning: 2% vs. 0.8% in suicide attempts, 1.8% vs. 1.4% in abuse, and 0.31% vs. 0.15% in accidental exposure. In the latter two circumstances, males also accounted for most notifications. Global suicide mortality is more than twice as high among men as among women (12.8 vs. 5.4 per 100,000) [15]. Masculinity norms centered on emotional restriction and the pursuit of status have been associated with suicidal behavior, particularly suicidal acts, which may contribute to this difference [16]. Conversely, the higher frequency of suicide attempts among women may be related, among other factors, to the higher prevalence of depressive disorders in this group, also observed in the Brazilian population [17].

Among women, the predominance of medications as the toxic agent is consistent with the self-medication profile reported by a Brazilian poison information center, where 62.8% of poisoning cases occurred among females, with anxiolytics and analgesics among the most frequent drug classes [4]. Agricultural pesticides, although much less frequent, showed the highest case fatality (211/6,324; 3.3%) in the present study. The profile of agricultural pesticide poisoning described in the literature shows a predominance of men with low educational attainment, exposed mainly in occupational or accidental circumstances [18]. In Brazil, of 1,309 deaths from occupational pesticide poisoning between 2000 and 2009, 51.9% occurred among agricultural workers, revealing an important occupational risk [19]. This combination of greater clinical severity and occupational exposure calls for special attention to rural workers.

Suicide attempt was the predominant circumstance in all age groups except children under 10 years, among whom accidental exposure accounted for 30,043 of 38,899 cases (77.2%). This pattern is consistent with Brazilian pediatric findings: in a toxicology referral hospital in Belo Horizonte, 83% of poisonings in children and adolescents resulted from accidental oral ingestion, concentrated among children under four years of age [20]. The exploratory behavior characteristic of early childhood, including the oral exploration of objects, contributes to this vulnerability [21].

RRAS 12 (São José do Rio Preto/Araçatuba) had the highest incidence rate in the state, four times higher than that of the RRAS with the lowest rate, with a statistically significant difference compared with the other 17 regions. This region comprises municipalities with intense agricultural production. An association between the volume of pesticides used and the occurrence of poisoning has been demonstrated in Brazilian municipalities with the highest agricultural production, allowing priorities to be identified for the surveillance of pesticide use [22]. Pesticide poisoning is known to be underreported in Brazil, even in states with the highest pesticide sales, including São Paulo [18]. Therefore, the contribution of agricultural exposures to the incidence observed in RRAS 12 may be underestimated. On the other hand, differences in notification practices among regions may also contribute to the heterogeneity observed, since more active surveillance may result in higher recorded rates. However, the presence of CIATox does not appear to explain the regional distribution on its own: RRAS hosting these services [5] include both the region with the highest rate (RRAS 12) and one of those with the lowest (RRAS 13, Barretos/Franca/Ribeirão Preto; 42.4 per 100,000 inhabitants per year), whereas some RRAS without CIATox, such as RRAS 10 and 14, had some of the highest rates.

São Paulo exceeded Brazil in all three overall indicators: incidence, mortality, and case fatality. The state not only follows the national pattern of increasing notifications and deaths from exogenous poisoning described between 2007 and 2019 [6] but does so at a greater magnitude. The higher density of CIATox in São Paulo, together with urbanization and broader access to medications, may favor both exposure and the capture of cases by the surveillance system. Part of the increase in notifications over the period may also reflect changes in surveillance rather than in the occurrence of poisoning, including the implementation of compulsory notification in August 2010, which makes the first segment of the series (2010-2012) particularly sensitive to the expansion of reporting, and progressive changes in surveillance practices, diagnostic awareness, access to health services, and reporting.

The decrease in notifications observed in 2020, both in São Paulo and in Brazil, coincides with the COVID-19 pandemic and may be related to underreporting during this period. In contrast, a study of exogenous poisoning notifications in the state of Bahia concluded that the pandemic did not significantly affect the notification profile, although the frequency of cases involving drugs of abuse increased [23]. Other studies have shown decreases in notifications of other conditions during the pandemic in Brazil and in São Paulo, such as tuberculosis cases and deaths [24,25].

The divergence between the sustained increase in incidence and the behavior of mortality and case fatality after 2016 stands out in the trend analysis. Incidence showed an upward trajectory throughout the period, whereas mortality increased mainly between 2010 and 2016 and became stationary from 2016 onwards. This stabilization contrasts with the national series of deaths from intentional self-poisoning by medications recorded in the Mortality Information System, which showed a marked increase from 2016 onwards [26]. This divergence may be explained by differences in data sources, since SINAN captures only notified cases, and in the scope of the outcomes analyzed. Case fatality did not follow the increase in incidence: with no significant trend over the entire period, it decreased between 2016 and 2020 in the main analysis, although this decline was not statistically significant in the sensitivity analyses and should be interpreted with caution. This relationship partly results from the stabilization of mortality in this period, but it may also reflect an improvement in the clinical response to identified cases. The CIATox network in the state, with specialized telephone support for real-time therapeutic and antidotal guidance, may have contributed to this improvement, although the study design does not allow causality to be established. Alternatively, the decline in case fatality may reflect the increasing notification of less severe cases, which enlarges the denominator: between 2016 and 2019, notifications in São Paulo almost doubled (from 26,217 to 50,929), whereas deaths did not increase (from 425 to 360). Incomplete outcome recording may also have contributed, since the proportion of notifications with missing outcome increased in the same period.

Underreporting in SINAN is not exclusive to exogenous poisoning but is a structural feature of the national surveillance system. A study comparing cases recorded in SINAN and in the Poison Information Center of the Federal District between 2009 and 2013 showed that the two systems complement each other: children up to nine years of age accounted for 53% of the cases captured by the poison center but only 33.9% of those in SINAN, suggesting a substantial loss of pediatric exposures in formal notification [27]. In a municipality in the state of Pará, a comparison between raw SINAN records and data consolidated in DATASUS from 2019 to 2023 revealed an average loss of 42% of notifications during consolidation, with losses of up to 91% for some conditions, the largest discrepancies being observed for acquired immunodeficiency syndrome (AIDS), syphilis in pregnant women, and dengue [28]. These findings suggest that underreporting results both from incomplete case capture by health services and from losses during data consolidation; therefore, the figures presented here should be interpreted as a minimum estimate of the problem.

This study has strengths, including the use of population-based data covering all notifications in the most populous Brazilian state over 11 years and the analysis by RRAS, which provides information applicable to regional health planning.

This study has limitations. First, it used secondary SINAN data, which are subject to underreporting and to incomplete data in investigation forms. The degree of underreporting in São Paulo could not be estimated, because comparable poison center or hospital records were not available for this study. Some variables had high proportions of missing data, such as education (n=124,400; 39.5%), which precluded their detailed analysis. The outcome was missing in 64,772 (20.5%) notifications; therefore, mortality and case fatality are likely underestimated, and the decline in case fatality after 2016 may be partly due to incomplete outcome recording. Second, as an ecological study, its findings refer to populations and should not be extrapolated to individuals. Third, the study period ended in 2020, the first year of the COVID-19 pandemic, which may have influenced the last point of the series. Analyses including more recent years are needed to assess post-pandemic trends. Fourth, municipalities were grouped according to the 18-RRAS configuration in force at the time of data extraction. In 2025, the separation of the Araçatuba region from São José do Rio Preto created a 19th RRAS [29].

## Conclusions

This study characterized the epidemiological profile of exogenous poisoning notifications in the state of São Paulo between 2010 and 2020, showing predominantly female exposure and greater severity among males, with medications as the main agent and suicide attempt as the most frequent circumstance. The highest incidence rate was concentrated in a region of intense agricultural activity, distinct from the capital, which had the highest absolute number of cases. Compared with Brazil, São Paulo showed higher incidence, mortality, and case fatality, with a faster increase in notifications.

Incidence increased throughout the period. Although the main joinpoint model indicated a decrease in case fatality after 2016, this finding was not statistically significant in sensitivity analyses and should therefore be interpreted cautiously. The observed pattern may partly reflect the increasing notification of less severe cases and incomplete outcome recording. Given the underreporting inherent to surveillance data, these figures likely underestimate the real magnitude of the problem. These results support prioritizing toxicological surveillance, suicide prevention strategies, and the control of medication and pesticide use in the state and reinforce the usefulness of regionalization by RRAS as a public health planning tool.
